# Reclassification of the Taxonomic Framework of Orders *Cellvibrionales, Oceanospirillales, Pseudomonadales*, and *Alteromonadales* in Class *Gammaproteobacteria* through Phylogenomic Tree Analysis

**DOI:** 10.1128/mSystems.00543-20

**Published:** 2020-09-15

**Authors:** Hu Liao, Xiaolan Lin, Yuqian Li, Mingming Qu, Yun Tian

**Affiliations:** a State Key Laboratory of Marine Environmental Science and Key Laboratory of the Ministry of Education for Coastal and Wetland Ecosystems, School of Life Sciences, Xiamen University, Xiamen, China; b Key Laboratory of Urban Environment and Health, Institute of Urban Environment, Chinese Academy of Sciences, Xiamen, China; c University of Chinese Academy of Sciences, Beijing, China; University of Copenhagen

**Keywords:** class *Gammaproteobacteria*, core genome, *Moraxellales* ord. nov., *Kangiellales* ord. nov., *Marinobacteraceae* fam. nov., *Zooshikellaceae* fam. nov., *Perlucidibacaceae* fam. nov., *Pseudomonadales* ord. nov.

## Abstract

Orders *Oceanospirillales* and *Pseudomonadales* play important roles in various ecosystems as the keystone taxa of microbiomes. However, the two orders present a close evolutionary relationship, which might have caused taxonomic misinterpretation and resulted in an incorrect understanding of their evolutionary history. In this study, first, we used the 16S rRNA gene sequences of 2,049 species of *Gammaproteobacteria* to build a phylogenetic tree, which demonstrated that reports regarding the evolutionary relationship of orders *Cellvibrionales*, *Oceanospirillales*, and *Pseudomonadales* based on a single conserved gene with a poor resolution have been conflicting; in particular, the major families *Moraxellaceae* and *Pseudomonadaceae* of order *Pseudomonadales* were separated from orders *Cellvibrionales* and *Oceanospirillales*. Subsequently, we constructed the bac120 trees of all representative reference genomes of class *Gammaproteobacteria* based on 120 ubiquitous single-copy proteins from bacteria and a phylogenomic tree based on the 119 core genes of 257 reference genomes obtained from orders *Cellvibrionales*, *Oceanospirillales*, and *Pseudomonadales* to cross validate and infer their intrinsic evolutionary relationships. These results indicated that two novel orders, *Moraxellales* ord. nov. and *Kangiellales* ord. nov., and three novel families, *Marinobacteraceae* fam. nov., *Perlucidibacaceae* fam. nov., and *Zooshikellaceae* fam. nov., should be proposed. Additionally, orders *Cellvibrionales* and *Oceanospirillales* were merged into the order *Pseudomonadales* except for families *Moraxellaceae* and *Kangiellaceae* in class *Gammaproteobacteria*, which currently includes 18 families. Our work sheds some light on the evolutionary history of class *Gammaproteobacteria*, which could facilitate the detection and taxonomic analysis of natural communities.

**IMPORTANCE** The orders *Cellvibrionales*, *Oceanospirillales*, and *Pseudomonadales*, as three major orders of the largest bacterial class, *Gammaproteobacteria*, play important roles in various ecosystems as the keystone taxa of microbiomes, but their evolutionary relationship is currently polyphyletic and chaotic. Here, we constructed a bac120 tree and core-genome tree and calculated the amino acid identity (AAI) value to explore their intrinsic evolutionary history. In this study, we proposed two novel orders and three novel families. This evolution study vastly reconstructed the taxonomic framework of class *Gammaproteobacteria* and could provide a more distinct perspective on global distribution and evolutionary patterns of these environmental microorganisms.

## Abstract (precis)

The orders *Cellvibrionales*, *Oceanospirillales*, and *Pseudomonadales*, as three major orders of the largest bacterial class, *Gammaproteobacteria*, play important roles in various ecosystems as the keystone taxa of microbiomes, but their evolutionary relationship is currently polyphyletic and chaotic. Here, we constructed a bac120 tree and core-genome tree and calculated the amino acid identity (AAI) value to explore their intrinsic evolutionary history. In this study, we proposed two novel orders and three novel families. This evolution study vastly reconstructed the taxonomic framework of class *Gammaproteobacteria* and could provide a more distinct perspective on global distribution and evolutionary patterns of these environmental microorganisms.

## INTRODUCTION

The tree of life is arguably the most important organizing principle in biology and perhaps the most widely understood depiction of the evolutionary process. It explains how we are related to other organisms and where we may have come from (1). With the continuous reduction in sequencing costs and new developments in biotechnology and bioinformatic tools, a multigene-based phylogenomic tree approach in which genomic data are used for phylogenomic analysis appears to be a better approach for defining genera or higher taxa than the use of 16S rRNA gene-derived phylogeny (2). In August 2018, Parks et al. (3) proposed a standardized bacterial taxonomy based on a genome phylogeny and substantially revised the tree of life. We believe that the new classification framework will provide important guidance for future reclassification studies.

*Gammaproteobacteria* spreads throughout global ecosystems, including marine, land, and sediment environments and animal hosts, and represents the largest bacterial class, including 19 orders, 58 families, and 381 genera according to LPSN and the website https://www.ezbiocloud.net/taxonomy?tn=Gammaproteobacteria&depth=3. To date, 78,338 *Gammaproteobacteria* genomes have been deposited in the National Center for Biotechnology Information (NCBI) database (October 2019); the representative genomes in the RefSeq category include 808 genomes. Therefore, phylogenomic tree construction based on all genomes of *Gammaproteobacteria* is difficult due to the restriction of computing resources; alternatively, the construction of an evolutionary tree based the representative genomes in the RefSeq category is relatively easy and accurate.

*Cellvibrionales*, *Oceanospirillales*, and *Pseudomonadales* are three major orders of *Gammaproteobacteria* that play important roles in various ecosystems as the keystone taxa of microbiomes (4). For instance, order *Cellvibrionales* has a putative important function in oligotrophic marine environments (5). The order *Oceanospirillales* shows remarkable potential for the natural attenuation of spilled oil in deep-sea surface sediments (6), and almost all species of family *Endozoicomonadaceae* have been isolated from marine animals, while most members of genus *Zooshikella* can produce prodigiosin, which is an effective proapoptotic agent that can be used against various cancer cell lines while showing little or no toxicity toward normal cell lines (7). The order *Pseudomonadales* plays an important role in contaminated soil remediation and plant-associated microbiota (4), and many members of order *Pseudomonadales* present clear associations with human health as pathogens, such as Acinetobacter baumannii, Moraxella catarrhalis, and Pseudomonas aeruginosa.

As of the writing of the manuscript, order *Oceanospirillales* includes 11 families (https://lpsn.dsmz.de/order/oceanospirillales), order *Pseudomonadales* includes 3 families (https://lpsn.dsmz.de/order/pseudomonadales), and order *Cellvibrionales* includes 5 families (5). In the last decade, based on rapid advances in phylogenetic and molecular analyses, several revisions have been carried out in the order *Oceanospirillales*, with numerous genera being split into separate families (5); for instance, family *Endozoicomonadaceae* was split from family *Hahellaceae* in 2018 (8). Additionally, genus *Marinobacterium* was reclassified into family *Oceanospirillaceae* (9), indicating that the systematic evolution of the order is still unclear due to the discovery of increasing numbers of species. In 2017, we discovered a novel genus, *Mangrovitalea*, which is closely related to genus *Marinobacter*, and we classified this new genus into order *Alteromonadales* (10). However, in this study, we found that genera *Tamilnaduibacter* (11), *Mangrovitalea*, and *Marinobacter* formed a robust clade in a phylogenetic tree and that they were distantly phylogenetically related to *Alteromonadales*; therefore, they should be allocated to higher taxonomic ranks. The major families *Moraxellaceae* and *Pseudomonadaceae* of order *Pseudomonadales* were separated by a branch containing orders *Cellvibrionales* and *Oceanospirillales* according to the 16S rRNA-based The All-Species Living Tree (LTP), release 132, which illustrated that the order *Pseudomonadales* had polyphyletic lineages. Intriguingly, in 2018, the Genome Taxonomy Database (GTDB) taxonomy proposed the transfer of the majority of the members of orders *Oceanospirillales* and *Cellvibrionales* to order *Pseudomonadales*; however, this classification has not been proposed anywhere in the literature, and thus, the intrinsic evolutionary relationship of orders *Oceanospirillales*, *Cellvibrionales*, and *Pseudomonadales* is still a question worth discussing.

## RESULTS AND DISCUSSION

### Phylogenetic tree based on small-subunit (SSU)-rRNA of *Gammaproteobacteria*.

**FIG 1:**
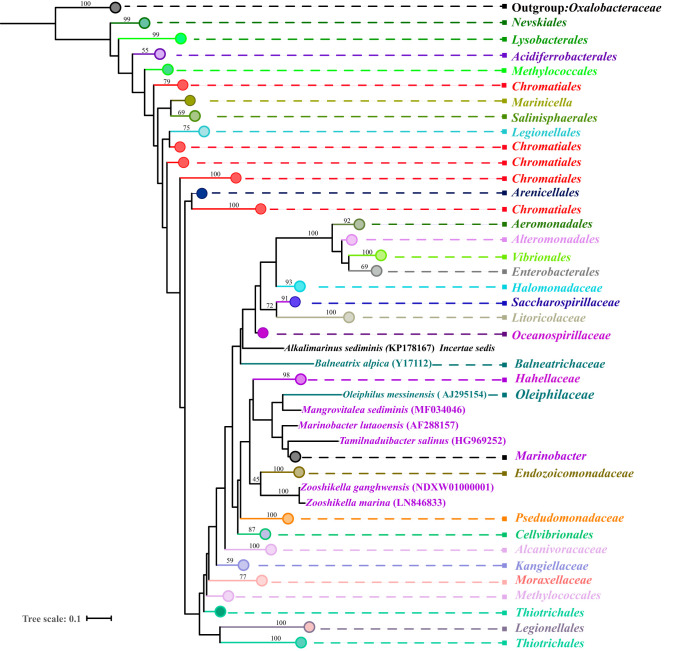
Maximum-likelihood tree showing the phylogenetic evolution of class *Gammaproteobacteria*. The phylogeny was inferred from 16S rRNA gene sequences of almost all type strains of *Gammaproteobacteria* in a maximum-likelihood framework by using RAxML with the GTRGAMMA model. Family *Oxalobacteraceae* was used as the outgroup of the tree. Bootstrap values are based on 1,000 replicates, and only bootstrap values greater than 40 are shown.

Figure 1 was generated based on 16S rRNA gene sequences, and 2,049 sequences representing 2,049 species of class *Gammaproteobacteria* with validly published names were downloaded from the SILVA Living Tree Project v128 database and the EzBioCloud database. The results indicated that genera *Marinobacter*, *Mangrovitalea*, and *Tamilnaduibacter* formed a monophyletic clade with family *Oleiphilaceae* (Fig. 1), which was also shown by the GTDB phylogeny reconstructed from 120 ubiquitous single-copy protein-coding genes (3), and these genera were distantly related to order *Alteromonadales*, implying that the monophyletic clade could represent a novel family. We refer to the clade as group 1 in the following text.

According to the phylogenetic tree based on 16S rRNA genes in group 1, *Zooshikella*, *Endozoicomonadaceae*, *Pseudomonadaceae*, and *Cellvibrionales* presented an indication of sharing a relatively close ancestor, whereas *Pseudomonadaceae* and *Moraxellaceae* were separated on different branches (Fig. 1). However, the topological structure of the branch with low bootstrap values (Fig. 1) indicated that the tree was unstable; therefore, it was also unclear what order group 1 belongs to in the tree.

### Evolutionary analysis based on the genomes.

**FIG 2:**
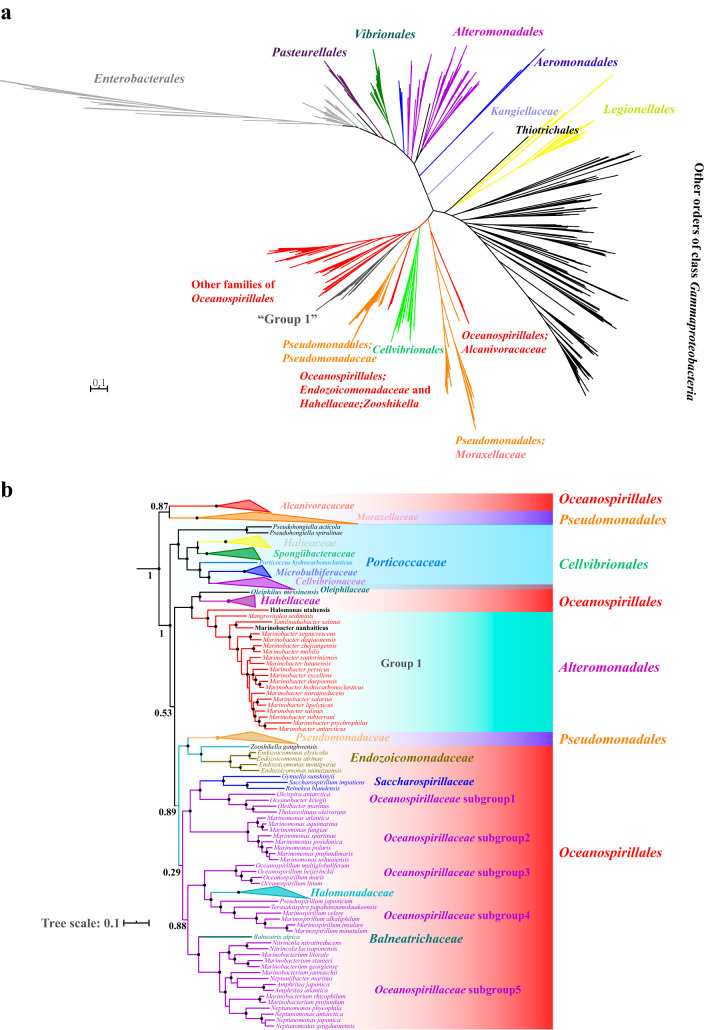
The unrooted maximum-likelihood tree was constructed by using FastTree with the WAG+CAT model based on 120 concatenated protein amino acid sequences of the 783 genomes. Each tip represents a species. (a) A pruned subtree from the unrooted maximum-likelihood tree. The bootstrap value of the backbone is displayed with a number. (b) Bootstrap values (from 0.9 to 1) are shown with filled circles. The tree was modified and visualized using the Interactive Tree of Life (iTOL 4.3) (itol.embl.de/).

We constructed a bac120 tree based on 120 concatenated ubiquitous single-copy proteins of bacteria (12) (Fig. 2; see also Fig. S1 in the supplemental material) from a total of 783 genomes (completeness >90% and contamination <5%) (Table S1) by using FastTree software according to the method described by Parks et al. (3). In 2018, the GTDB taxonomy proposed the transfer of the majority of the members of *Oceanospirillales* and *Cellvibrionales* and group 1 to *Pseudomonadales*, and *Kangiellaceae* was transferred to order *Enterobacterales* (3). Similarly, orders *Pseudomonadales*, *Cellvibrionales*, and *Oceanospirillales* and group 1 were also clustered on a branch with the support of the highest bootstrap value of 1.0, except for family *Kangiellaceae* in class *Gammaproteobacteria*, and according to Fig. 2a and Fig. S1, the family *Kangiellaceae* formed an independent branch at the order level that was different from GTDB taxonomy. In addition, the clade of family *Moraxellaceae* displayed the longest length in Fig. 2b and was located away from other families of order *Pseudomonadales*; Fig. 2b indicated they were partitioned by the branch of the order *Cellvibrionales*.

10.1128/mSystems.00543-20.1 FIG S1 The uncollapsed tree from Fig. 2a provides all clade information. Download FIG S1, PDF file, 1.5 MB. Copyright © 2020 Liao et al. 2020 Liao et al. This content is distributed under the terms of the Creative Commons Attribution 4.0 International license.

10.1128/mSystems.00543-20.8 TABLE S1 Main information for the genome sequences used to reconstruct the bac120 tree. Download Table S1, XLSX file, 0.1 MB. Copyright © 2020 Liao et al. 2020 Liao et al. This content is distributed under the terms of the Creative Commons Attribution 4.0 International license.

Then, we chose 257 reference genomes of *Pseudomonadales*, *Cellvibrionales*, *Oceanospirillales*, and group 1 for further analysis; the major information for these genomes is collected in Table S2. The pangenomes of the reference genomes were analyzed, and the results indicated that they shared 119 core genes, which were annotated according to the UniProt database (13); these proteins are mostly involved in DNA replication, transcription and translation, and ATP production. The sizes of the core and pangenomes were strongly dependent on the number of genomes analyzed, resulting in shrinking core genomes and expanding pangenomes with an increase in the depth of genome sampling (Fig. S2).

10.1128/mSystems.00543-20.2 FIG S2 Core–pangenome size evolution of the 257 reference genomes. Pangenome size (green) is directly proportional to the number of genomes. Core-genome size (yellow) is inversely proportional to the number of genomes. Download FIG S2, DOCX file, 0.4 MB. Copyright © 2020 Liao et al. 2020 Liao et al. This content is distributed under the terms of the Creative Commons Attribution 4.0 International license.

10.1128/mSystems.00543-20.9 TABLE S2 Main information for the 257 reference genome sequences used to reconstruct the core-genome tree. Download Table S2, XLSX file, 0.05 MB. Copyright © 2020 Liao et al. 2020 Liao et al. This content is distributed under the terms of the Creative Commons Attribution 4.0 International license.

**FIG 3:**
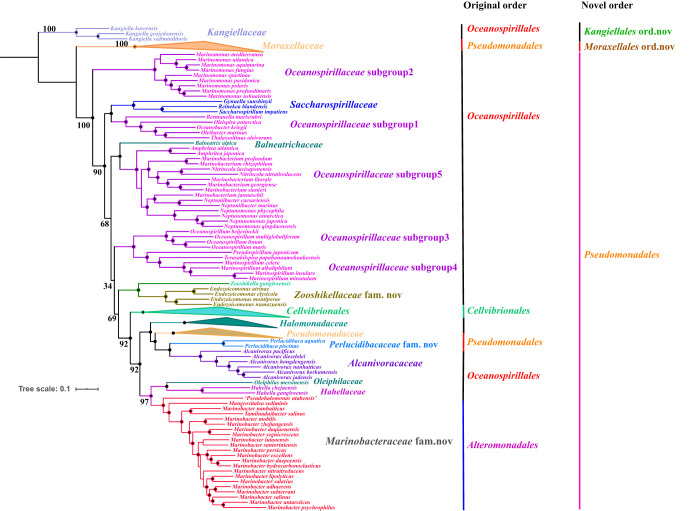
Maximum-likelihood tree constructed by using IQtree with the optimal model based on the concatenated core-genome sequences of the 257 genomes. Bootstrap values (expressed as percentages of 1,000 replicates) greater than 90 are shown at branch points with filled circles, and the bootstrap value of the backbone is displayed with a number. The tree was modified and drawn using the Interactive Tree of Life (iTOL 4.3) (itol.embl.de/).

Subsequently, a core-genome-based phylogeny was reconstructed based on 119 concatenated single-copy core genes of the 257 genomes with optimal models by using the IQtree package. The results showed that the topological structure of the tree based on the core genome (Fig. 3 and Fig. S3) was highly similar to the bac120 tree (Fig. 2b and Fig. S1).

10.1128/mSystems.00543-20.3 FIG S3 The uncollapsed tree from Fig. 3 provides all clade information. Download FIG S3, PDF file, 0.4 MB. Copyright © 2020 Liao et al. 2020 Liao et al. This content is distributed under the terms of the Creative Commons Attribution 4.0 International license.

**FIG 4:**
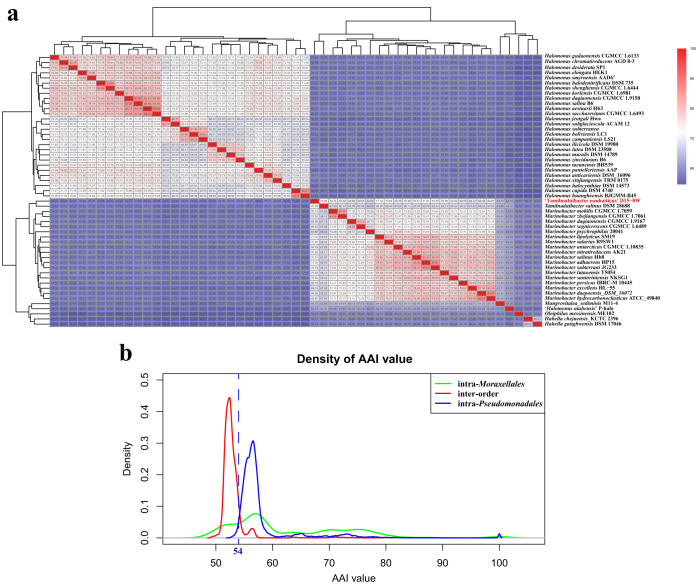
(a) Heatmap showing the AAI values between genera *Halomonas*, *Marinobacter*, *Mangrovitalea*, and *Tamilnaduibacter*. One species in red, *Marinobacter nanhaiticus*, was renamed “*Tamilnaduibacter nanhaiticus*” in the present study, respectively. (b) Density of the AAI values of intra-*Moraxellales* (green line); the interorder (red line) between orders *Moraxellales* and *Pseudomonadales*; and intra-*Pseudomonadales* (blue line). The names of the orders were proposed in the study (blue line) (b).

The amino acid identity (AAI) values between the 257 genomes were calculated as well because AAI values are used for prokaryotic taxonomic analyses (14). The AAI comparisons conducted by Luo et al. (15) indicated that related but different genera typically exhibit values ranging from 60% to 80%; thus, interfamilies typically exhibit values of less than 60%. In our study, the AAI values were clustered in a heatmap via the complete method of hclust (Fig. 4); we found that the interfamily AAI values were below 60% and that intrafamily AAI values were mostly greater than 60% (Fig. S4), consistent with the work of Luo et al. (15).

10.1128/mSystems.00543-20.4 FIG S4 Heatmap showing the AAI values between the 257 genomes. Download FIG S4, PDF file, 1.2 MB. Copyright © 2020 Liao et al. 2020 Liao et al. This content is distributed under the terms of the Creative Commons Attribution 4.0 International license.

The order *Oceanospirillales* was paraphyletic (Fig. 2a and Fig. 3). The type genus *Kangiella* (16) of family *Kangiellaceae* (17) formed a stably separate clade, was positioned away from order *Oceanospirillales*, and was distinct from closely related orders *Aeromonadales* (18) and *Thiotrichales* (19) of class *Gammaproteobacteria* based on the bac120 tree with bootstrap value 100 (Fig. 2a and Fig. S1). In terms of physiological phenotypic characteristics, the genomic G+C content of family *Kangiellaceae* ranged from 40.1 to 44.4%, whereas that of *Oceanospirillales* ranged from 43.1 to 68.6%, revealing a significant difference (Wilcoxon test; *P* < 0.01). Additionally, extracellular protein degradation and amino acid utilization are significant and prominent features of the type genus *Kangiella* of family *Kangiellaceae* due to the absence of a complete pathway for carbohydrate metabolism according to the description of Wang et al. (20); for instance, almost all members of genus *Kangiella* can hydrolyze casein and gelatin, while most of the members of order *Oceanospirillales* were negative for that (Table 1). In terms of fatty acid composition characteristics, the major fatty acid components of family *Kangiellaceae* are iso-C_15:0_, C_16:0_ 10-methy, and iso-C_11:0_ 3-OH (21), which are obviously different from almost all other members of order *Oceanospirillales*, in which C_16:0_, C_16 : 1_ *ω*7*c*, and/or C_16 : 1_ *ω*6*c* are the major fatty acid components, The major polar lipids of almost all members of family *Kangiellaceae* were phosphatidylglycerol (PG), phosphatidylethanolamine (PE), and phosphatidylmonomethylethanolamine (PME) (22), while those for the order *Oceanospirillales* were diphosphatidylglycerol (DPG), PE, and PG. Additionally, Q-8 was the predominant ubiquinone of family *Kangiellaceae*, while the predominant ubiquinone is Q-9 in all other members of order *Oceanospirillales*. A Manhattan-based principal-coordinate analysis (PCoA) of the gene presence and absence profile also showed that the members of the type genus *Kangiella* of family *Kangiellaceae* formed a cluster divided from other genera of order *Oceanospirillales* (Fig. S5). These evidences indicated that family *Kangiellaceae* should be reclassified as the novel order *Kangiellales* ord. nov., including the family *Kangiellaceae*, of which the type genus is *Kangiella*. Despite the GTDB classifying the order *Kangiellales* into the order *Enterobacterales* (23, 24), however, the order *Enterobacterales* was very large, including some clades with excessive branch length based on the bac120 tree (Fig. S1 and Fig. 2a). Additionally, the major fatty acids of almost all members of order *Enterobacterales* were C_14:0_, C_16:0_, C_18:1_ *ω*7*c*, and C_17:0_ (24), illustrating an obvious difference from order *Kangiellales*. Therefore, we inferred the classification was inaccurate in the GTDB.

**TABLE 1:** Phenotypic characteristics of *Kangiellales*, *Moraxellales*, *Pseudomonadales*, *Enterobacterales*, and *Perlucidibacaceae*^*a*^

	*Kangiellales*	*Moraxellales*	*Pseudomonadales*	*Enterobacterales*	*Perlucidibacaceae*
Cell shape	Rods	Short rods, coccoid, or coccal	Rods, spiral	Rods	Rods
G+C content (%)	40.1–44.4	38–48	43.1–68.6	22–60	55–65
Fatty acids	iso-C_15:0_, C_16:0_ 10-methy, and iso-C_11:0_ 3-OH	C_18:1_ *ω*9*c*, C_18:0_, C_16:0_, and C_16:1_ ω6*c*/C_16:1_ ω7*c*	C_16:0_, C_16 : 1_ *ω*7*c*, and/or C_16 : 1_ *ω*6*c*	C_14:0_, C_16:0_, C_16:1_ *ω*7*c*	C_16:0_, C_18:1_ *ω*7*c*, C_16 : 1_ *ω*7*c* and/or C_16 : 1_ *ω*6*c*, and C_12:0_ 3-OH
Ubiquinone	Q8	Q8	Q9	NA	Q12
Flagellation	+	−	+	+	+
Hydrolysis of:					
Casein	+	NA	V	NA	NA
Gelatin	+	NA	V	NA	NA

a The data are from original isolation papers and/or *Bergey’s Manual*. References are as follows: *Kangiellales*, 17; *Moraxellales*, 29; *Pseudomonadales*, 30, 36; *Enterobacterales*, 30. The names of the orders or family were proposed in the study. NA, not applicable; +, present/tested positive; −, absent/tested negative; V, variable among strains.

10.1128/mSystems.00543-20.5 FIG S5 Manhattan distance-based principal-coordinate analysis (PCoA) showing the difference in the gene presence and absence profiles between different genera. Download FIG S5, PDF file, 0.7 MB. Copyright © 2020 Liao et al. 2020 Liao et al. This content is distributed under the terms of the Creative Commons Attribution 4.0 International license.

Intriguingly, the shared gene blocks of *Pseudomonadaceae* (including genera *Azotobacter*, *Pseudomonas*, and *Oblitimonas*) and *Moraxellaceae* (including genera Acinetobacter, *Alkanindiges*, *Moraxella*, *Perlucidibaca* [25], and *Psychrobacter*) displayed obvious distinction, and the dendrogram of heatmap rows revealed that they formed an independent branch that was consistent with the topology of the bac120 and core-genome tree (Fig. 3 and Fig. S3 and S6). Additionally, the type genus *Ventosimonas* of family *Ventosimonadaceae* formed a clade within family *Pseudomonadaceae* with a long branch length in the bac120 and core-genome tree (Fig. S1 and S5). We also observed that *Alcanivoracaceae*, *Balneatrichaceae*, *Halomonadaceae*, *Hahellaceae*, *Oleiphilaceae*, *Oceanospirillaceae*, *Saccharospirillaceae*, *Zooshikellaceae*, *Pseudomonadaceae*, *Ventosimonadaceae*, *Cellvibrionaceae*, *Halieaceae*, *Microbulbiferaceae*, *Porticoccaceae*, *Spongiibacteraceae*, and group 1 shared more genes with each other than they shared with *Moraxellaceae* (Fig. S6). A Manhattan-based principal-coordinate analysis (PCoA) of the gene presence and absence profile also showed that the members of the type genus *Pseudomonas* of family *Pseudomonadeae* and the type genus *Moraxella* of family *Moraxellaceae* clustered in different quadrants (Fig. S6). Additionally, the phenotypic characteristics between family *Moraxellaceae* and other families of order *Pseudomonadales* are notably different. First, almost all members of family *Moraxellaceae* contain C_18:1_ *ω*9*c* as a major fatty acid component (26, 27), while the component was not detected in other families of *Pseudomonadales* (28). Second, the cell shapes of family *Moraxellaceae* are short rods or coccoid or coccal or may exhibit a characteristic multicellular micromorphology, and cells usually occur in pairs or short chains (29); however, other families of order *Pseudomonadales* have just one cell form that is rod-shaped, and cells usually occur in singles (Table 1). Third, the cells of family *Moraxellaceae* are nonmotile in liquid media and do not exhibit flagellation, but the other families of order *Pseudomonadales* typically have polar flagella (Table 1) (30). Fourth, except for some strains of Acinetobacter and *Psychrobacter*, no acid is produced from carbohydrates in family *Moraxellaceae*; however, the other families of order *Pseudomonadales* can produce acid from glucose and so on (30). In addition, the genome size and G+C% between *Pseudomonadaceae* and *Moraxellaceae* present significant differences (Wilcoxon test; *P* < 0.0001) (Fig. S7). In light of these results, it is proposed that *Moraxella* be reclassified as the type genus of *Moraxellales* ord. nov.

10.1128/mSystems.00543-20.6 FIG S6 Heatmap of the gene presence and absence profiles of the 257 reference genomes. The numeral 1 represents the presence of genes, and 0 represents the absence of genes. Download FIG S6, TIF file, 2.7 MB. Copyright © 2020 Liao et al. 2020 Liao et al. This content is distributed under the terms of the Creative Commons Attribution 4.0 International license.

10.1128/mSystems.00543-20.7 FIG S7 Genomic G+C% of families *Pseudomonadaceae* and *Moraxellaceae* (a). Genome size of families *Pseudomonadaceae* and *Moraxellaceae* (b). Download FIG S7, DOCX file, 0.1 MB. Copyright © 2020 Liao et al. 2020 Liao et al. This content is distributed under the terms of the Creative Commons Attribution 4.0 International license.

**FIG 5:**
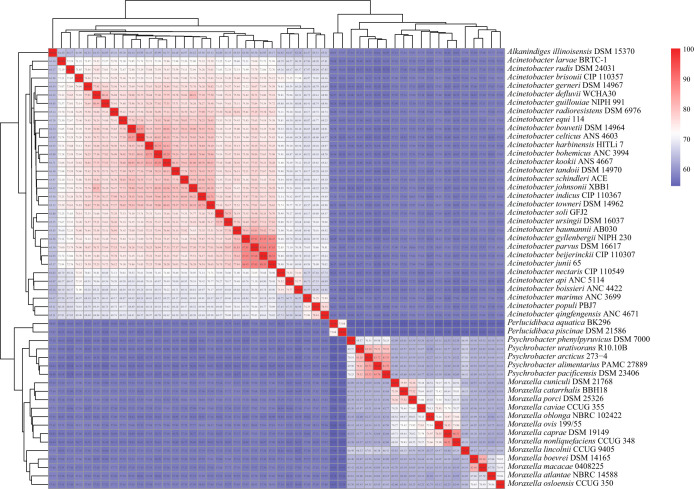
Heatmap showing the AAI values between genera Acinetobacter, *Alkanindiges*, *Moraxella*, *Psychrobacter*, and *Perlucidibaca*.

However, we found the family *Moraxellaceae* was paraphyletic and formed three separate clades, and the three clades clustered with genera *Moraxella* and *Psychrobacter*, *Alkanindiges* and Acinetobacter, and *Perlucidibaca* in the bac120 and core-genome tree, respectively (Fig. 3 and Fig. S1 and S5). The genus *Perlucidibaca* was positioned away from the other two clades in the core-genome tree (Fig. 3 and Fig. S6). The comparison of the AAI values, and shared gene blocks of the pangenome of intrafamily *Moraxellaceae*, also indicated that genus *Perlucidibaca* apparently differed from genera Acinetobacter, *Alkanindiges*, *Moraxella*, and *Psychrobacter* (Fig. 5 and Fig. S4). In addition, the phenotypic synapomorphies of *Perlucidibaca* obviously differed from those of Acinetobacter, *Alkanindiges*, *Moraxella* (25), and *Psychrobacter*. For example, the original description of *Perlucidibaca* (25) indicated that the members of this taxon are facultatively aerobic and that their anaerobic growth is similar to aerobic growth, whereas genera Acinetobacter, *Alkanindiges*, *Psychrobacter*, and *Moraxella* are strictly aerobic bacteria (25). Besides, the major fatty acids of genus *Perlucidibaca* are C_16:0_, C_18:1_ *ω*7*c*, C_16 : 1_ *ω*7*c*, and/or C_16 : 1_ *ω*6*c* and C_12:0_ 3-OH, while C_18:1_ *ω*9*c* is a minor component (31), the major respiratory quinone of genus *Perlucidibaca* is Q-12 (32), while that of the major member of family *Moraxella* is Q-8, and additionally, the cells of genus *Perlucidibaca* usually occur as singles (Table 1) (31). In light of these results, it is proposed that *Perlucidibaca* should be reclassified as the type genus of *Perlucidibacaceae* fam. nov., and it should be shifted out of the novel order *Moraxellales* and merged into order *Pseudomonadales*.

As shown in Fig. 3, group 1 formed a robust lineage and presented a close relationship with families *Hahellaceae* and *Oleiphilaceae* in the bac120 and core-genome tree (Fig. 2 and 3), and the GTDB taxonomy classified the branch as belonging to family *Oleiphilaceae*; however, we found that the G+C content of the genera *Marinobacter*, *Mangrovitalea*, and *Tamilnaduibacter* ranged from 53.7 to 63.2% and that of family *Oleiphilaceae* ranged from 43.4 to 47.8%. Additionally, the AAI value between group 1, *Hahellaceae*, and family *Oleiphilaceae* was less than 60% (Fig. 4a). Furthermore, many species of group 1 can utilize various carbon sources including aliphatic and polycyclic aromatic hydrocarbons, acyclic isoprenoid compounds, and many sole carbon sources, while all strains of family *Oleiphilaceae* can use only aliphatic hydrocarbons and their derivatives as carbon sources for growth (33). Additionally, the cellular fatty acid patterns of most strains of group 1 were C_16: 0_, C_18:1_ *ω*9*c*, C_16:1_ *ω*7*c*, and/or C_16 : 1_ *ω*6*c* and C_12:0_ 3-OH, while those of the *Oleiphilaceae* were C_16: 0_, C_16:1_ *ω*7*c*, and/or C_16 : 1_ *ω*6*c* and C_16:1_ *ω*9*c*. The major polar lipids of group 1 were diphosphatidylglycerol (DPG), phosphatidylethanolamine (PE), and phosphatidylglycerol (PG), and those of the family *Oleiphilaceae* were PE, PG, and phosphatidyldimethylethylamine (DME) (33); thus, these chemotaxonomic indices between group 1 and family *Oleiphilaceae* have certain differences. These results indicated that the two clades represented two different families, contradicting the GTDB taxonomy. Therefore, we designated the lineage as family *Marinobacteraceae* fam. nov. because the first valid name of the genus of this clade was *Marinobacter*, first proposed in 1992 (34); the family comprises four genera: *Marinobacter*, *Mangrovitalea*, *Pseudohalomonas*, and *Tamilnaduibacter*. In addition, the species “*Marinobacter nanhaiticus*” (35) was transferred from genus *Marinobacter* to *Tamilnaduibacter* and named “*Tamilnaduibacter nanhaiticus*” comb. nov., which was suggested because the species “*Marinobacter nanhaiticus*” always forms a robust clade with genus *Tamilnaduibacter* in the bac120 tree and the core-genome tree (Fig. 2 and 3), and the AAI value between Tamilnaduibacter salinus and “*T. nanhaiticus*” was 70.99 and higher than those of *T. salinus* and other *Marinobacter* members (Fig. 4a).

*Oceanospirillaceae* (36) appeared to be polyphyletic and formed five highly supported subgroups in the bac120 and core-genome tree, implying that it should be split into five novel families. However, *Oceanospirillaceae* has been found to include 21 genera (https://www.ezbiocloud.net/taxonomy?tn=Oceanospirillaceae&depth=2) thus far, whereas only 15 genera were obtained in the core-genome tree. Hence, additional genome sequences need to be made available if these new families are to be proposed.

We also observed that the family *Endozoicomonadaceae* and the genus *Zooshikella* formed a robust clade with family *Pseudomonadaceae*, indicating that the genus *Zooshikella* presents the closest evolutionary relationship with family *Endozoicomonadaceae* (Fig. 2 and 3 and Fig. S1). The family *Endozoicomonadaceae* and genus *Zooshikella* shared more genes with each other than they shared with other families (Fig. S6). Additionally, the phenotypical synapomorphies of *Zooshikella* and family *Endozoicomonadaceae* are not obviously different; for example, they have similar major fatty acid components including C_16:0_, C_18 : 1_ ω7*c*, C_16:1_ *ω*7*c*, and/or C_16:1_ *ω*6*c*, the major quinone was Q-9, and almost all members of genus *Zooshikella* and family *Endozoicomonadaceae* were mesophilic (37, 38), except that genus *Endozoicomonas* also included C_10:0_ 3-OH as a major hydroxyl fatty acid component; further, PE, PG, phosphatidylserine (PS), and DPG are present in the polar lipid pattern of family *Endozoicomonadaceae*, while the genus *Zooshikella* shows DPG, PE, and PG, except that PS was not detected (Table 2) (8, 39). In light of these results, the family *Endozoicomonadaceae* should be transferred to a novel family and named *Zooshikellaceae* fam. nov. because the genus *Zooshikella* was first proposed in 2003 within the clade (37), and the family comprises four genera: *Endozoicomonas*, *Kistimonas*, *Parendozoicomonas* (8), and *Zooshikella*. The family name has been given already (https://gtdb.ecogenomic.org/searches?q=%25Zooshikella%25&s=al), but the classification is different from GTDB in that family *Zooshikellaceae* includes only the genus *Zooshikella*. This proposal does not conflict with the 16S rRNA gene tree provided in the initial description of the genus, despite the tree being poorly resolved.

**TABLE 2:** Phenotypic characteristics of *Marinobacteraceae* fam. nov., *Oleiphilaceae*, *Zooshikellaceae*, and “*Endozoicomonadaceae*”^*a*^

	*Marinobacteraceae*	*Oleiphilaceae*	*Zooshikellaceae*	“*Endozoicomonadaceae*”
Colony color	White	NA	Red	Beige
Fatty acids	C_16: 0_, C_18:1_ *ω*9*c*, C_16:1_ *ω*7*c* and/or C_16 : 1_ *ω*6*c*, and C_12:0_ 3-OH	C_16: 0_, C_16:1_ *ω*7*c* and/or C_16 : 1_ *ω*6*c*, and C_16:1_ *ω*9*c*	C_16:0_, C_16:1_ *ω*7*c* and/or C_16:1_ *ω*6*c*, and C_18 : 1_ *ω*7*c*	C_16:0_, C_16:1_ *ω*7*c* and/or C_16:1_ *ω*6*c*, C_18 : 1_ *ω*7*c*, and C_10:0_ 3-OH
Polar lipids	DPG, PE, PG	PE, PG, DME	DPG, PG, PE	PE, PG, PS, DPG
Ubiquinone	Q9	Q9	Q9	Q9
G+C content (%)	53.7–63.2	43.4–47.8	40.2–41.3	47.0–51.0
Genomic size (Mbp)	3.4–5.3	6.4	5.8	5.4–6.7

a The data are from original isolation papers and/or *Bergey’s Manual*. References are as follows: *Marinobacteraceae* fam. nov., 34; *Oleiphilaceae*, 33; *Zooshikellaceae*, 37; “*Endozoicomonadaceae*,” 8. The names of the families were proposed in the study, except “*Endozoicomonadaceae*.” NA, not applicable; +, present/tested positive; −, absent/tested negative; V, variable among strains.

As indicated by the results presented in Fig. S4 and Fig. 4b, the AAI values between *Alcanivoracaceae*, *Balneatrichaceae*, *Halomonadaceae*, *Hahellaceae*, *Oleiphilaceae*, *Oceanospirillaceae*, *Saccharospirillaceae*, *Zooshikellaceae*, *Pseudomonadaceae*, *Perlucidibacaceae*, *Cellvibrionaceae*, *Halieaceae*, *Microbulbiferaceae*, *Porticoccaceae*, *Spongiibacteraceae*, *Ventosimonadaceae*, and *Marinobacteraceae* were significantly higher than those between the above taxa and *Moraxellaceae* (Wilcox test *P* < 0.0001). Together, the results from this study indicated that 17 families shared a common ancestor at the order level; therefore, these families were merged into one order, *Pseudomonadales*, consistent with the designation of the GTDB (3) because the first species proposed was Pseudomonas aeruginosa in 1900. The closest order to *Pseudomonadales* is *Moraxellales*. A possible AAI threshold of 54 was proposed to differentiate among orders according to Fig. 4b, but it needs further study to be used in other complex phyla such as *Firmicutes* or *Bacteroidetes*.

## DISCUSSION

### Emendation of the order *Pseudomonadales*.

In this study, we proved that two novel families, *Marinobacteraceae* and *Perlucidibacaceae*; families *Alcanivoracaceae* (40), *Balneatrichaceae*, *Halomonadaceae* (41), *Hahellaceae* (42), *Oleiphilaceae* (33), *Oceanospirillaceae*, *Saccharospirillaceae* (43), and *Zooshikellaceae* of order *Oceanospirillales*; *Pseudomonadaceae* and *Ventosimonadaceae* (28) of order *Pseudomonadales*; and *Cellvibrionaceae*, *Halieaceae*, *Microbulbiferaceae*, *Porticoccaceae*, and *Spongiibacteraceae* (5) of order *Cellvibrionales* shared a relatively recent ancestor and formed a robust branch based on two typical phylogenomic tree and AAI values in class *Gammaproteobacteria*. The family *Litoricolaceae* should also be classified into order *Pseudomonadales* tentatively because it formed a stable clade with family *Saccharospirillaceae* (bootstrap value 72) based on the 16S rRNA tree, as no genome is available for *Litoricolaceae*. In addition, the family *Natronospirillaceae* was proposed by Kevbrin et al. (43), and when we submitted the article to the journal in 2020, the closest family to family *Natronospirillaceae* was *Saccharospirillaceae* according to the description by Kevbrin et al. (43); thus, the family *Natronospirillaceae* should be classified into order *Pseudomonadales* as well. Therefore, orders *Oceanospirillales*, *Cellvibrionales*, and *Pseudomonadales* were merged into the single order *Pseudomonadales* with the exception of families *Moraxellaceae* and *Kangiellaceae*, including 19 families in the partial taxonomic reconstruction of class *Gammaproteobacteria*.

Almost all members of order *Pseudomonadales* are mesophilic, the major fatty acid components are C_16:0_, C_16:1_ *ω*7*c* and/or C_16:1_ *ω*6*c*, and C_18:1_ *ω*7*c*, and the major respiratory quinone is Q-9.

Type genus: *Pseudomonas*; class: *Gammaproteobacteria*.

### Description of *Moraxellales* ord. nov.

*Moraxellales* (Mo.ra.xel.la′les. N.L. fem. dim. n. *Moraxella* type genus of the order; suff. -ales, ending denoting an order; N.L. fem. pl. n. *Moraxellales*, the *Moraxella* order).

The description is the same as that for family *Moraxellaceae* (29). Type genus: *Moraxella*; class: *Gammaproteobacteria*.

### Description of *Kangiellales* ord. nov.

*Kangiellales* (Kan.gi.el.la.les. N.L. fem. dim. n. *Kangiella*, type genus of the order; suff. -ales, ending denoting an order; N.L. fem. pl. n. *Kangiellales*, the *Kangiella* order).

The description is the same as that for family *Kangiellaceae* (17). Type genus: *Kangiella*; class: *Gammaproteobacteria*.

### Description of *Marinobacteraceae* fam. nov.

*Marinobacteraceae* (Ma.ri.no.bac.te.ra′ce.ae. N.L. masc. n. *Marinobacter*, type genus of the family; -aceae, suff. ending denoting a family; N.L. fem. pl. n. *Marinobacteraceae*, the *Marinobacter* family).

The family belongs to order *Oceanospirillales*, class *Gammaproteobacteria*, and mainly consists of bacteria isolated from the sediments of marine environments. The cellular fatty acid patterns of most strains are C_16: 0_, C_18:1_ *ω*9*c*, summed features 3 and C_12:0_ 3-OH. The G+C content of the genomic DNA ranges from 53.7 to 63.2%. At present, the family comprises genera *Marinobacter*, *Mangrovitalea*, *Pseudohalomonas*, and *Tamilnaduibacter*. The definition of the family relies mainly on the construction of phylogenetic relationships based on 16S rRNA gene sequences and phylogenomic relationships based on core genomes and concatenated 120 ubiquitous single-copy protein sequences.

Type genus: *Marinobacter*; order: *Pseudomonadales*.

### Description of *Perlucidibacaceae* fam. nov.

*Perlucidibacaceae* (Per.lu.ci.di.ba.ca'ce.ae. N.L. fem. n. *Perlucidibaca*, type genus of the family; -aceae, suff. ending denoting a family; N.L. fem. pl. n. *Perlucidibacaceae*, the *Perlucidibaca* family).

The description is the same as for genus *Perlucidibaca* (25, 32).

Type genus: *Perlucidibaca*; order: *Pseudomonadales*.

### Description of *Zooshikellaceae* fam. nov.

*Zooshikellaceae* (Zoo.shi′ke.lla′ce.ae. N.L. fem. dim. n. *Zooshikella*, type genus of the family; -aceae, suff. ending denoting a family; N.L. fem. pl. n. *Zooshikellaceae*, the *Zooshikella* family).

The major fatty acid components were C_16:0_, C_16:1_ *ω*7*c* and/or C_16:1_ *ω*6*c*, and C_18:1_ *ω*7*c*; the major quinone was Q-9; PE, PG, PS, and DPG are present in the major polar lipid pattern; and almost all members of the family were mesophilic. At present, the family comprises genera *Endozoicomonas*, *Kistimonas*, *Parendozoicomonas*, and *Zooshikella*. Members of this family form a stable clade in the reconstructed phylogenetic tree based on 16S rRNA gene sequences and the phylogenomic tree based on core genomes and concatenated 120 ubiquitous single-copy protein sequences. The type genus of the family is *Zooshikella*.

## MATERIALS AND METHODS

### SSU-rRNA-based phylogeny.

Reference sequences of class *Gammaproteobacteria* with valid published names were downloaded from the SILVA Living Tree Project v128 database and the EzBioCloud database. The package MAFFT v7.402 was used for sequence alignment, and identical sequences were deleted by using RAxML before constructing the tree. Phylogenetic trees based on data sets of 16S rRNA gene sequences were constructed using RAxML (44) by applying the -f a, -p 12345, -x 12345, -# 1,000 or 200, and -m GTRGAMMA parameters. The 16S rRNA gene identity values were obtained through a BLASTN all-versus-all sequence similarity search.

### Reference genome of *Gammaproteobacteria*.

First, 808 reference genomes of *Gammaproteobacteria* out of 78,338 genomes were downloaded from the genome database of the NCBI on 19 October 2019. Then, the genomes of the species Mangrovitalea sediminis (PRJNA402051) and Tamilnaduibacter salinus (PRJNA442664) were added to the data set. Then, genome completeness and contamination were controlled by using CheckM, and the genomes exhibiting <90% completeness or >5% contamination were filtered out. Finally, 783 high-quality genomes were obtained for subsequent analysis. The major information for the genomes is listed in Table S1 in the supplemental material, and genome size ranged from 0.3 Mbp to 7.8 Mbp.

### Phylogenetic analysis of 120 ubiquitous single-copy proteins.

The bac120 tree was inferred from the dereplicated data set by applying the WAG model (45) of protein evolution with gamma-distributed rate heterogeneity (46) (+GAMMA) in FastTree to a concatenated alignment of 120 ubiquitous single-copy proteins (12) with the GTDB -tk tool (3). These trees were modified and visualized using the Interactive Tree of Life (iTOL) (itol.embl.de/).

### Pangenome and phylogenomic analysis.

The sequences were annotated using Prokka v1.12 (47). The pangenome was estimated using the rapid large-scale prokaryotic pangenome analysis (Roary v3.11.2) tool (48) with parameters -i 50 -cd 99. Briefly, the annotated genes from all 257 representative reference genomes of orders *Oceanospirillales*, *Cellvibrionales*, and *Pseudomonadales* and genera *Tamilnaduibacter*, *Mangrovitalea*, and *Marinobacter* (Table S2) were first filtered to remove partial sequences and iteratively preclustered with CD-HIT. These procedures resulted in a substantially reduced set of protein sequences. An all-against-all comparison of the reduced sequences with 50% sequence identity was performed with BLASTP. The sequences were then clustered with Markov clustering algorithm (MCL), and the preclustering results from CD-HIT were finally merged together with the results of MCL. Homologous clusters were divided into core, accessory, and unique genomes. The core genome comprised genes shared within at least 99% of the genomes. The cumulative sizes of the pangenome and core genome were calculated by selecting genomes with replacement in random order 500 times and then calculating the mean size of each sampling point.

A pangenome matrix was generated based on the presence or absence of all genetic loci in each individual genome produced by Roary. We selected the top 6,000 genes of the matrix to produce the heatmap with the pheatmap package, species were clustered, and PCoA was performed based on the presence and absence of orthologs according to the Manhattan distance by using hclust in R.

### Phylogenomic analysis of 119 concatenated single-copy core genes.

The phylogenomic tree was generated based on the concatenated single-copy core genes. The core-genome sequences were accurately aligned with MAFFT v7.402. The resulting multiple sequence alignment length was 121,975 bp and retained 37,526 bp after trimming with Gblocks 0.91b (49) with default paraments. A phylogenomic tree was inferred using the IQtree package to search optimal models and further verify the morphologies and topologies of the phylogenomic tree (50) using the command -bb 1000 -m MFP+MERGE+R, and the RAxML program was applied with the parameters -f a, -p 12345, -x 12345, -# 200, and -m GTRGAMMAI (50), based on trimmed concatenated single-copy core genes.

### Whole-genome relatedness indices.

The AAI is the mean amino acid identity of orthologous genes. To validate our taxonomic proposals, we performed AAI comparisons between these genomes. The AAI indices were deduced from pairwise conserved comparisons of coding proteins and calculated using CompareM v0.0.21 software (https://github.com/dparks1134/CompareM) (which employs DIAMOND v0.9.24 to obtain the best reciprocal hits [51]) with the default BLASTP parameters (i.e., 10−5 E value, 30% sequence identity cutoff, and ≥70% alignment length) to define the bidirectional best BLAST hits between genomes. The resulting AAI values were clustered by using hclust, and a heatmap was generated with the pheatmap package in R.

### Data processing and availability.

All data (codes, other supplemental tables, and files) are available at the website https://github.com/liaohu1231/phylogenomic_analysis.

## References

[1] Castelle CJ, Banfield JF. 2018. Major new microbial groups expand diversity and alter our understanding of the tree of life. Cell 172:1181–1197. doi:10.1016/j.cell.2018.02.016. 29522741

[2] Chun J, Oren A, Ventosa A, Christensen H, Arahal DR, da Costa MS, Rooney AP, Yi H, Xu X-W, De Meyer S, Trujillo ME. 2018. Proposed minimal standards for the use of genome data for the taxonomy of prokaryotes. Int J Syst Evol Microbiol 68:461–466. doi:10.1099/ijsem.0.002516. 29292687

[3] Parks DH, Chuvochina M, Waite DW, Rinke C, Skarshewski A, Chaumeil P-A, Hugenholtz P. 2018. A standardized bacterial taxonomy based on genome phylogeny substantially revises the tree of life. Nat Biotechnol 36:996–1004. doi:10.1038/nbt.4229. 30148503

[4] Banerjee S, Schlaeppi K, van der Heijden MGA. 2018. Keystone taxa as drivers of microbiome structure and functioning. Nat Rev Microbiol 16:567–576. doi:10.1038/s41579-018-0024-1. 29789680

[5] Spring S, Scheuner C, Göker M, Klenk H-P. 2015. A taxonomic framework for emerging groups of ecologically important marine gammaproteobacteria based on the reconstruction of evolutionary relationships using genome-scale data. Front Microbiol 6:281. doi:10.3389/fmicb.2015.00281. 25914684 PMC4391266

[6] Bacosa HP, Erdner DL, Rosenheim BE, Shetty P, Seitz KW, Baker BJ, Liu Z. 2018. Hydrocarbon degradation and response of seafloor sediment bacterial community in the northern Gulf of Mexico to light Louisiana sweet crude oil. ISME J 12:2532–2543. doi:10.1038/s41396-018-0190-1. 29950702 PMC6154971

[7] Darshan N, Manonmani HK. 2015. Prodigiosin and its potential applications. J Food Sci Technol 52:5393–5407. doi:10.1007/s13197-015-1740-4. 26344956 PMC4554646

[8] Bartz JO, Blom J, Busse HJ, Mvie JB, Hardt M, Schubert P, Wilke T, Goessmann A, Wilharm G, Bender J, Kampfer P, Glaeser SP. 2018. *Parendozoicomonas haliclonae* gen. nov. sp. nov. isolated from a marine sponge of the genus Haliclona and description of the family *Endozoicomonadaceae* fam. nov. comprising the genera *Endozoicomonas*, *Parendozoicomonas*, and *Kistimonas*. Syst Appl Microbiol 41:73–84. doi:10.1016/j.syapm.2017.11.004. 29398077

[9] Satomi M, Fujii T. 2014. The family *Oceanospirillaceae*, p 491–527. *In* Rosenberg E, DeLong EF, Lory S, Stackebrandt E, Thompson F (ed), The prokaryotes: Gammaproteobacteria. Springer, Berlin, Germany.

[10] Liao H, Li Y, Guo X, Lin X, Lai Q, Xu H, Zheng T, Tian Y. 2017. *Mangrovitalea sediminis* gen. nov., sp. nov., a member of the family Alteromonadaceae isolated from mangrove sediment. Int J Syst Evol Microbiol 67:5172–5178. doi:10.1099/ijsem.0.002433. 29043950

[11] Verma A, Mual P, Mayilraj S, Krishnamurthi S. 2015. *Tamilnaduibacter salinus* gen. nov., sp. nov., a halotolerant gammaproteobacterium within the family *Alteromonadaceae*, isolated from a salt pan in Tamilnadu, India. Int J Syst Evol Microbiol 65:3248–3255. doi:10.1099/ijsem.0.000401. 26296662

[12] Parks DH, Rinke C, Chuvochina M, Chaumeil P-A, Woodcroft BJ, Evans PN, Hugenholtz P, Tyson GW. 2017. Recovery of nearly 8,000 metagenome-assembled genomes substantially expands the tree of life. Nat Microbiol 2:1533–1542. doi:10.1038/s41564-017-0012-7. 28894102

[13] Chen C, Huang H, Wu CH. 2017. Protein bioinformatics databases and resources, p 3–39. *In* Wu CH, Arighi CN, Ross KE (ed), Protein bioinformatics: from protein modifications and networks to proteomics. Springer, New York, NY.

[14] Rosselló-Mora R. 2005. Updating prokaryotic taxonomy. J Bacteriol 187:6255–6257. doi:10.1128/JB.187.18.6255-6257.2005. 16159756 PMC1236658

[15] Luo C, Rodriguez-R LM, Konstantinidis KT. 2014. MyTaxa: an advanced taxonomic classifier for genomic and metagenomic sequences. Nucleic Acids Res 42:e73. doi:10.1093/nar/gku169. 24589583 PMC4005636

[16] Yoon J-H, Oh T-K, Park Y-H. 2004. *Kangiella koreensis* gen. nov., sp. nov. and *Kangiella aquimarina* sp. nov., isolated from a tidal flat of the Yellow Sea in Korea. Int J Syst Evol Microbiol 54:1829–1835. doi:10.1099/ijs.0.63156-0. 15388751

[17] Wang G, Tang M, Wu H, Dai S, Li T, Chen C, He H, Fan J, Xiang W, Li X. 2015. *Aliikangiella marina* gen. nov., sp. nov., a marine bacterium from the culture broth of *Picochlorum* sp. 122, and proposal of *Kangiellaceae* fam. nov. in the order *Oceanospirillales*. Int J Syst Evol Microbiol 65:4488–4494. doi:10.1099/ijsem.0.000601. 26363841

[18] Martin-Carnahan A, Joseph SW. 2005 *Aeromonadales* ord. nov., p 556–587. *In* Brenner DJ, Krieg NR, Staley JT, Garrity GM, Boone DR, De Vos P, Goodfellow M, Rainey FA, Schleifer K-H (ed), Bergey’s manual of systematic bacteriology, vol 2. The Proteobacteria, part B. The Gammaproteobacteria. Springer, Boston, MA.

[19] Garrity GM, Bell JA, Lilburn T. 2005. *Thiotrichales* ord. nov., p 131–210. *In* Brenner DJ, Krieg NR, Staley JT, Garrity GM, Boone DR, De Vos P, Goodfellow M, Rainey FA, Schleifer K-H (ed), Bergey’s manual of systematic bacteriology, vol 2. The Proteobacteria, part B. The Gammaproteobacteria. Springer, Boston, MA.

[20] Wang J, Lu Y, Nawaz MZ, Xu J. 2018. Comparative genomics reveals evidence of genome reduction and high extracellular protein degradation potential in *Kangiella*. Front Microbiol 9:1224. doi:10.3389/fmicb.2018.01224. 29930545 PMC6000758

[21] Xu F-D, Li X-G, Xiao X, Xu J. 2015. *Kangiella profundi* sp. nov., isolated from deep-sea sediment. Int J Syst Evol Microbiol 65:2315–2319. doi:10.1099/ijs.0.000257. 25870256

[22] Lee SY, Park S, Oh TK, Yoon JH. 2013. Kangiella sediminilitoris sp. nov., isolated from a tidal flat sediment. Int J Syst Evol Microbiol 63:1001–1006. doi:10.1099/ijs.0.040691-0. 22685109

[23] Adeolu M, Alnajar S, Naushad S, Gupta RS. 2016. Genome-based phylogeny and taxonomy of the ‘*Enterobacteriales*’: proposal for Enterobacterales ord. nov. divided into the families *Enterobacteriaceae*, *Erwiniaceae* fam. nov., *Pectobacteriaceae* fam. nov., *Yersiniaceae* fam. nov., *Hafniaceae* fam. nov., *Morganellaceae* fam. nov., and *Budviciaceae* fam. nov. Int J Syst Evol Microbiol 66:5575–5599. doi:10.1099/ijsem.0.001485. 27620848

[24] Octavia S, Lan R. 2014. The family *Enterobacteriaceae*. *In* Rosenberg E, DeLong EF, Lory S, Stackebrandt E, Thompson F (ed), The prokaryotes. Springer, Berlin, Germany.

[25] Song J, Choo Y-J, Cho J-C. 2008. *Perlucidibaca piscinae* gen. nov., sp. nov., a freshwater bacterium belonging to the family *Moraxellaceae*. Int J Syst Evol Microbiol 58:97–102. doi:10.1099/ijs.0.65039-0. 18175691

[26] Bozal N, Montes MJ, Tudela E, Guinea J. 2003. Characterization of several *Psychrobacter* strains isolated from Antarctic environments and description of *Psychrobacter luti* sp. nov. and *Psychrobacter fozii* sp. nov. Int J Syst Evol Microbiol 53:1093–1100. doi:10.1099/ijs.0.02457-0. 12892132

[27] Humphreys GJ, Oates A, Ledder RG, McBain AJ. 2015. *Faucicola mancuniensis* gen. nov., sp. nov., isolated from the human oropharynx. Int J Syst Evol Microbiol 65:11–14. doi:10.1099/ijs.0.066837-0. 25267870

[28] Lin JY, Hobson WJ, Wertz JT. 2016. *Ventosimonas gracilis* gen. nov., sp. nov., a member of the *Gammaproteobacteria* isolated from Cephalotes varians ant guts representing a new family, *Ventosimonadaceae* fam. nov., within the order ‘*Pseudomonadales*’. Int J Syst Evol Microbiol 66:2869–2875. doi:10.1099/ijsem.0.001068. 27054961

[29] Rossau R, Van Landschoot A, Gillis M, De Ley J. 1991. Taxonomy of *Moraxellaceae* fam. nov., a new bacterial family to accommodate the genera *Moraxella*, *Acinetobacter*, and *Psychrobacter* and related organisms. Int J Syst Evol Microbiol 41:310–319. doi:10.1099/00207713-41-2-310.

[30] Brown DR, Tasker S, Messick JB, Neimark H. 2010. Bergey’s manual of systematic bacteriology. Springer, New York, NY.

[31] Baek K, Han J-H, Lee M-H. 2017. *Perlucidibaca aquatica* sp. nov., isolated from fresh water. Int J Syst Evol Microbiol 67:2296–2300. doi:10.1099/ijsem.0.001940. 28741994

[32] França L, Albuquerque L, da Costa MS. 2015. *Cavicella subterranea* gen. nov., sp. nov., isolated from a deep mineral-water aquifer, and emended description of the species *Perlucidibaca piscinae*. Int J Syst Evol Microbiol 65:3812–3817. doi:10.1099/ijsem.0.000493. 28875925

[33] Golyshin PN, Chernikova TN, Abraham W-R, Lünsdorf H, Timmis KN, Yakimov MM. 2002. *Oleiphilaceae* fam. nov., to include Oleiphilus messinensis gen. nov., sp. nov., a novel marine bacterium that obligately utilizes hydrocarbons. Int J Syst Evol Microbiol 52:901–911. doi:10.1099/00207713-52-3-901. 12054256

[34] Gauthier MJ, Lafay B, Christen R, Fernandez L, Acquaviva M, Bonin P, Bertrand JC. 1992. *Marinobacter hydrocarbonoclasticus* gen. nov., sp. nov., a new, extremely halotolerant, hydrocarbon-degrading marine bacterium. Int J Syst Bacteriol 42:568–576. doi:10.1099/00207713-42-4-568. 1382536

[35] Gao W, Cui Z, Li Q, Xu G, Jia X, Zheng L. 2013. *Marinobacter nanhaiticus* sp. nov., polycyclic aromatic hydrocarbon-degrading bacterium isolated from the sediment of the South China Sea. Antonie Van Leeuwenhoek 103:485–491. doi:10.1007/s10482-012-9830-z. 23117603

[36] Garrity GM, Bell JA, Lilburn T. 2005. *Oceanospirillales* ord. nov., p 270–323. *In* Brenner DJ, Krieg NR, Staley JT, Garrity GM, Boone DR, De Vos P, Goodfellow M, Rainey FA, Schleifer K-H (ed), Bergey’s manual of systematic bacteriology, vol 2. The Proteobacteria, part B. The Gammaproteobacteria. Springer, Boston, MA.

[37] Yi H, Chang Y-H, Oh HW, Bae KS, Chun J. 2003. *Zooshikella ganghwensis* gen. nov., sp. nov., isolated from tidal flat sediments. Int J Syst Evol Microbiol 53:1013–1018. doi:10.1099/ijs.0.02521-0. 12892120

[38] Yang C-S, Chen M-H, Arun AB, Chen CA, Wang J-T, Chen W-M. 2010. *Endozoicomonas montiporae* sp. nov., isolated from the encrusting pore coral Montipora aequituberculata. Int J Syst Evol Microbiol 60:1158–1162. doi:10.1099/ijs.0.014357-0. 19666790

[39] Ramaprasad EVV, Bharti D, Sasikala C, Ramana CV. 2015. *Zooshikella marina* sp. nov. a cycloprodigiosin- and prodigiosin-producing marine bacterium isolated from beach sand. Int J Syst Evol Microbiol 65:4669–4673. doi:10.1099/ijsem.0.000630. 26409875

[40] Golyshin PN, Harayama S, Timmis KN, Yakimov MM. 2005. Family II. *Alcanivoraceae* fam. nov., p 295. *In* Brenner DJ, Krieg NR, Staley JT, Garrity GM, Boone DR, De Vos P, Goodfellow M, Rainey FA, Schleifer K-H (ed), Bergey’s manual of systematic bacteriology, 2nd ed, vol 2. The Proteobacteria, part B. The Gammaproteobacteria. Springer, Boston, MA.

[41] Franzmann PD, Wehmeyer U, Stackebrandt E. 1988. *Halomonadaceae* fam. nov., a new family of the class *Proteobacteria* to accommodate the genera *Halomonas* and *Deleya*. Syst Appl Microbiol 11:16–19. doi:10.1016/S0723-2020(88)80043-2.

[42] Garrity GM, Bell JA, Lilburn T. 2015. *Hahellaceae* fam. nov. *In* Trujillo ME, Dedysh S, DeVos P, Hedlund B, Kämpfer P, Rainey FA, Whitman WB (ed), Bergey’s manual of systematics of archaea and bacteria. John Wiley & Sons, Inc, Hoboken, NJ.

[43] Kevbrin V, Boltyanskaya Y, Grouzdev D, Koziaeva V, Park M, Cho J-C. 2020. *Natronospirillum operosum* gen. nov., sp. nov., a haloalkaliphilic satellite isolated from decaying biomass of a laboratory culture of cyanobacterium Geitlerinema sp. and proposal of *Natronospirillaceae* fam. nov., *Saccharospirillaceae* fam. nov. and *Gynuellaceae* fam. nov. Int J Syst Evol Microbiol 70:511–521. doi:10.1099/ijsem.0.003781. 31671055

[44] Stamatakis A. 2006. RAxML-VI-HPC: maximum likelihood-based phylogenetic analyses with thousands of taxa and mixed models. Bioinformatics 22:2688–2690. doi:10.1093/bioinformatics/btl446. 16928733

[45] Whelan S, Goldman N. 2001. A general empirical model of protein evolution derived from multiple protein families using a maximum-likelihood approach. Mol Biol Evol 18:691–699. doi:10.1093/oxfordjournals.molbev.a003851. 11319253

[46] Yang Z. 1994. Maximum likelihood phylogenetic estimation from DNA sequences with variable rates over sites: approximate methods. J Mol Evol 39:306–314. doi:10.1007/BF00160154. 7932792

[47] Seemann T. 2014. Prokka: rapid prokaryotic genome annotation. Bioinformatics 30:2068–2069. doi:10.1093/bioinformatics/btu153. 24642063

[48] Page AJ, Cummins CA, Hunt M, Wong VK, Reuter S, Holden MT, Fookes M, Falush D, Keane JA, Parkhill J. 2015. Roary: rapid large-scale prokaryote pan genome analysis. Bioinformatics 31:3691–3693. doi:10.1093/bioinformatics/btv421. 26198102 PMC4817141

[49] Talavera G, Castresana J. 2007. Improvement of phylogenies after removing divergent and ambiguously aligned blocks from protein sequence alignments. Syst Biol 56:564–577. doi:10.1080/10635150701472164. 17654362

[50] Nguyen LT, Schmidt HA, von Haeseler A, Minh BQ. 2015. IQ-TREE: a fast and effective stochastic algorithm for estimating maximum-likelihood phylogenies. Mol Biol Evol 32:268–274. doi:10.1093/molbev/msu300. 25371430 PMC4271533

[51] Buchfink B, Xie C, Huson DH. 2015. Fast and sensitive protein alignment using DIAMOND. Nat Methods 12:59–60. doi:10.1038/nmeth.3176. 25402007

