# Supplementary material for: Reclassification of the Taxonomic Framework of Orders Cellvibrionales, Oceanospirillales, Pseudomonadales, and Alteromonadales in Class Gammaproteobacteria through Phylogenomic Tree Analysis
Source: mSystems. 2020 Sep 15;5(5):e00543-20. doi: 10.1128/mSystems.00543-20 (PMC7498684; doi:10.1128/mSystems.00543-20)
Supplement: FIG S1 [file mSystems.00543-20-sf001.pdf]

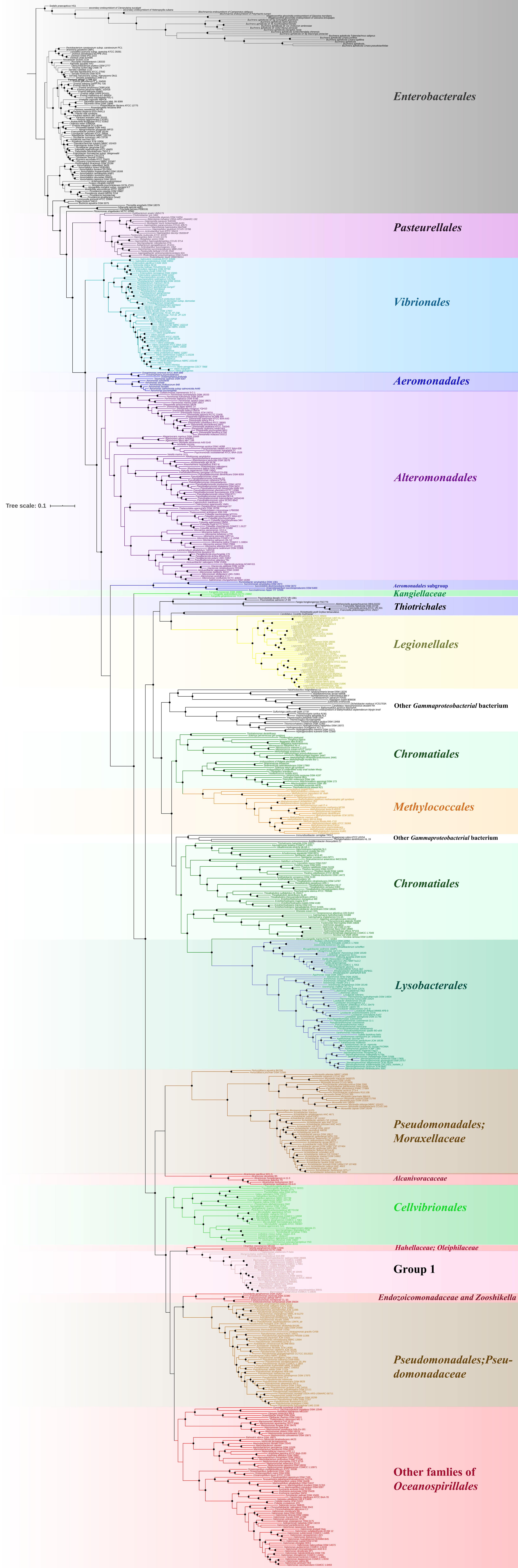

Tree scale: 0.1
Enterobacterales
Pasteurellales
Vibrionales
Aeromonadales
Alteromonadales
Aeromonadales subgroup
Kangiellaceae
Thiotrichales
Legionellales
Other Gammaproteobacterial bacterium
Chromatiales
Methylococcales
Other Gammaproteobacterial bacterium
Chromatiales
Lysobacterales
Pseudomonadales; Moraxellaceae
Alcanivoracaceae
Cellvibrionales
Hahellaceae; Oleiphilaceae
Group 1
Endozoicomonadaceae and Zooshikella
Pseudomonadales;Pseu-domonadaceae
Other families of Oceanospirillales
