# Supplementary figures and images for: Reclassification of the Taxonomic Framework of Orders Cellvibrionales, Oceanospirillales, Pseudomonadales, and Alteromonadales in Class Gammaproteobacteria through Phylogenomic Tree Analysis
Source: mSystems. 2020 Sep 15;5(5):e00543-20. doi: 10.1128/mSystems.00543-20 (PMC7498684; doi:10.1128/mSystems.00543-20)

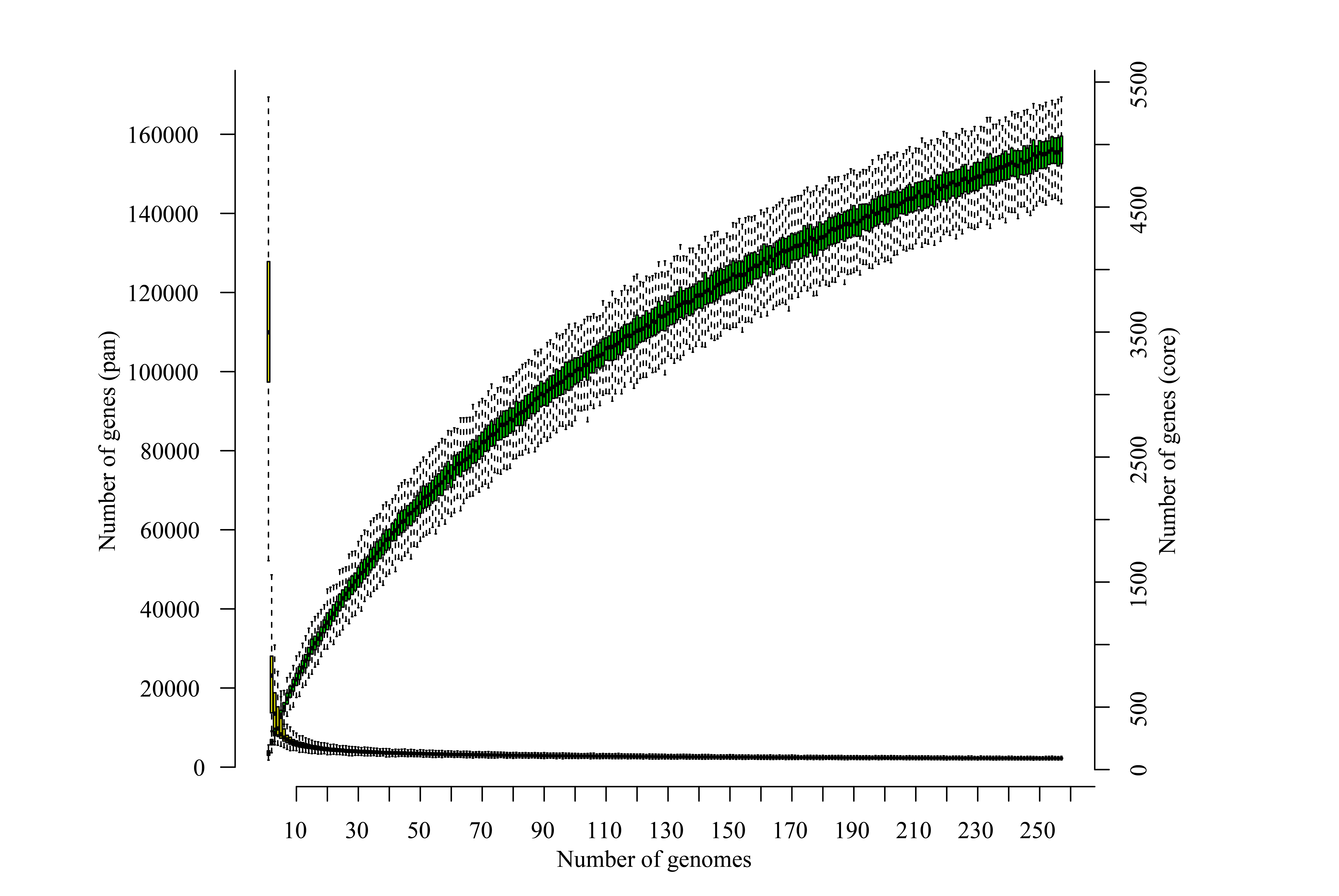


**Figure S2**

Supplement: FIG S2 [file mSystems.00543-20-sf002.docx]

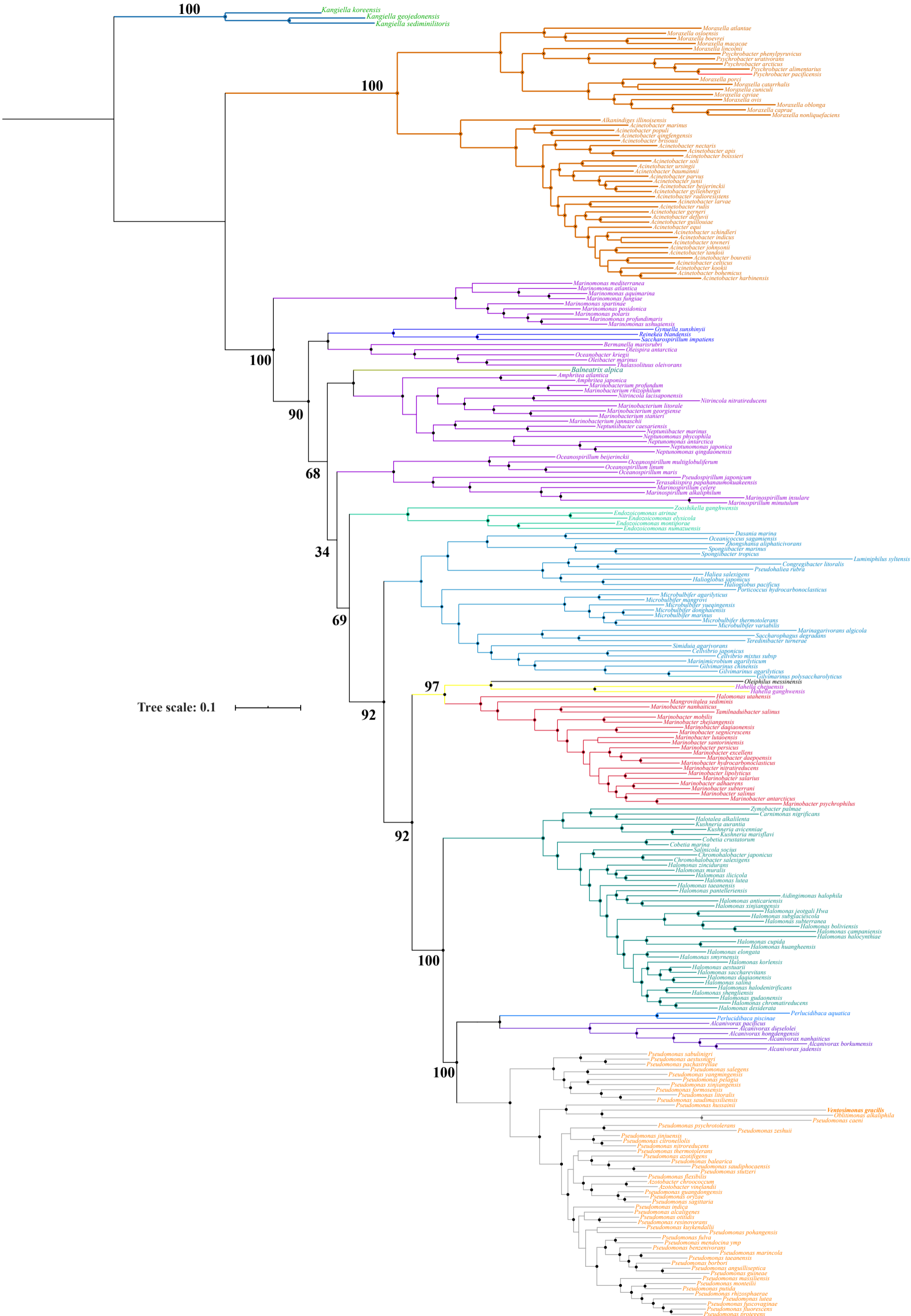

Supplement: FIG S3 [file mSystems.00543-20-sf003.pdf]

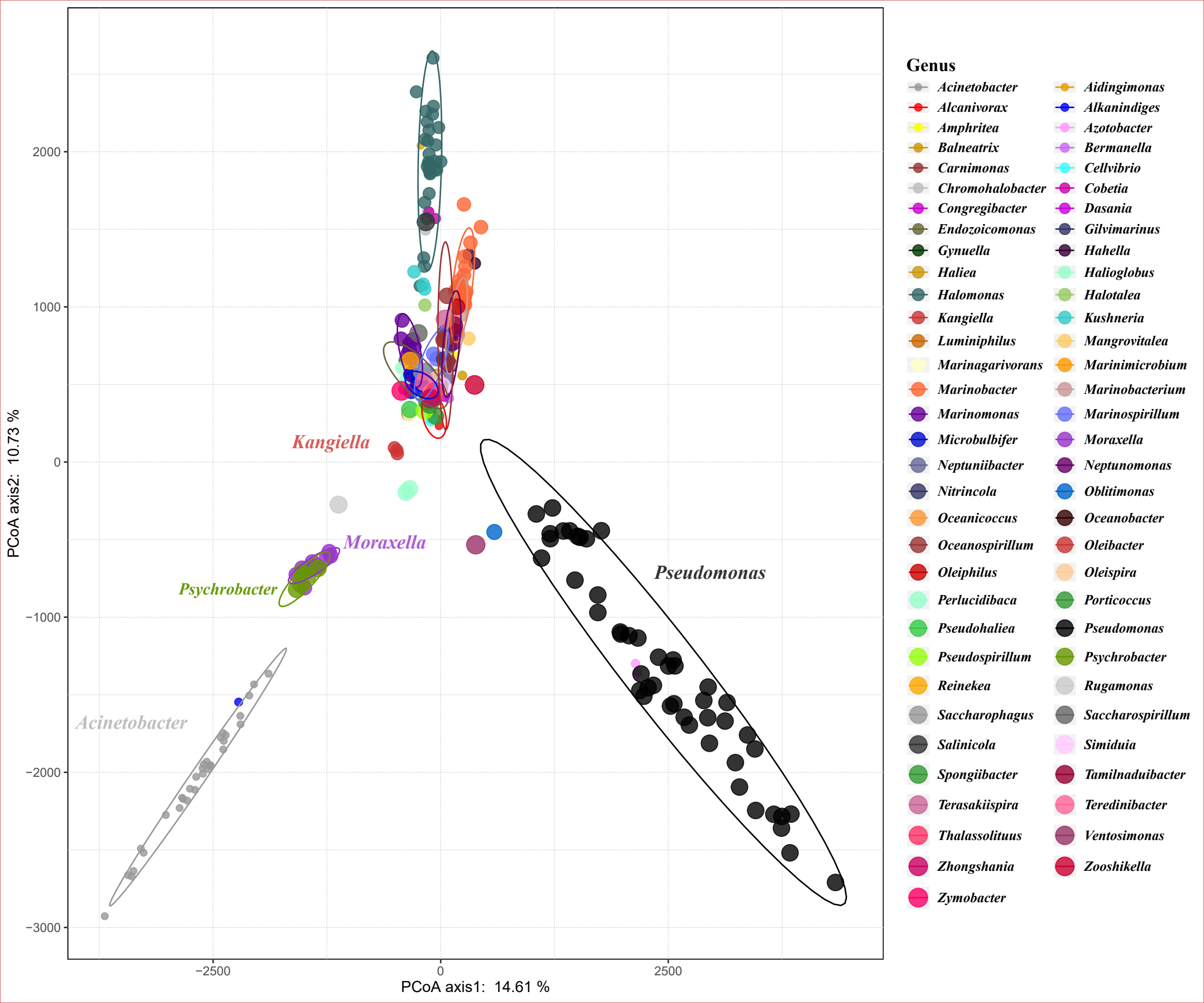

Supplement: FIG S5 [file mSystems.00543-20-sf005.pdf]

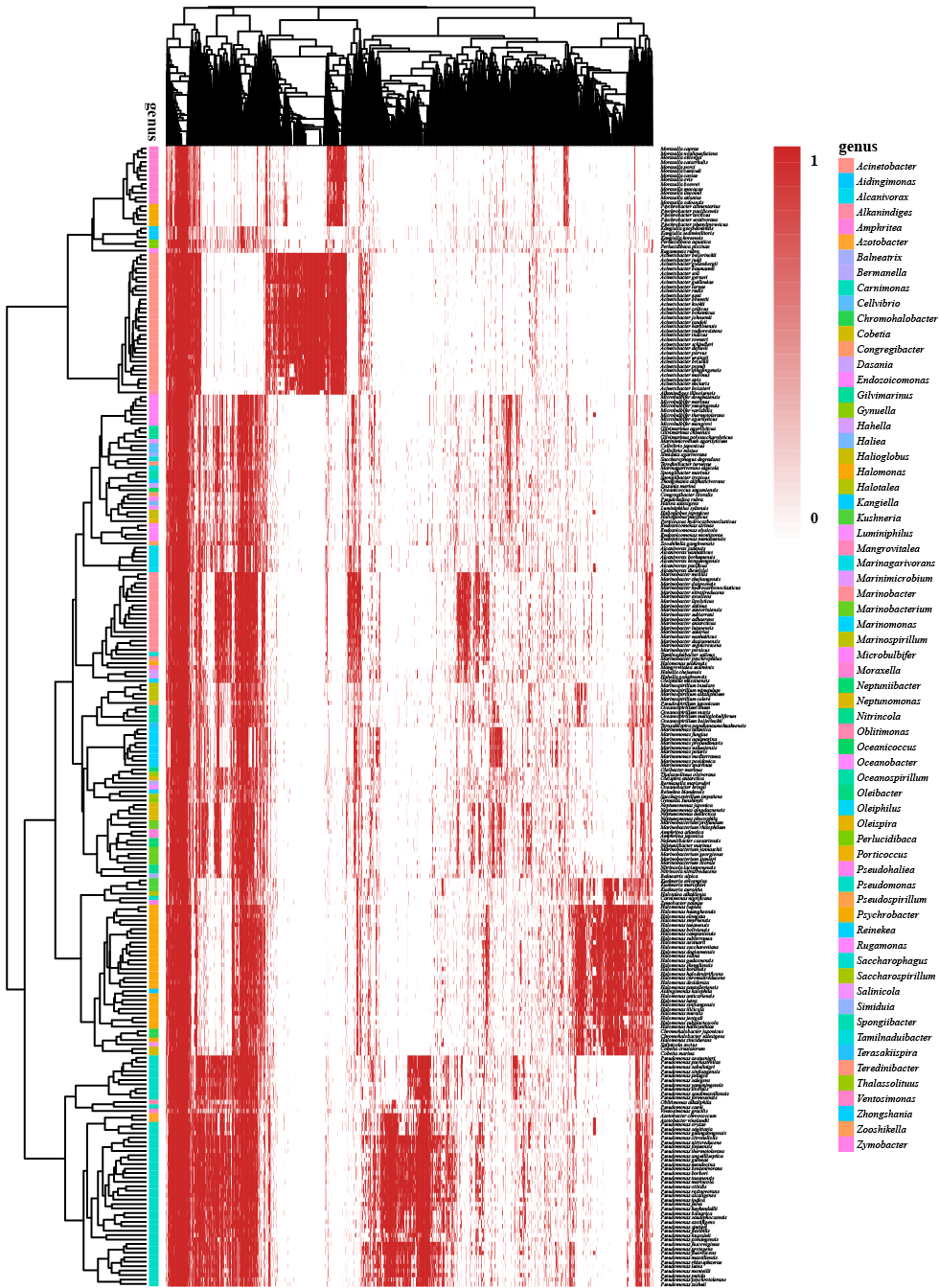

Supplement: FIG S6 [file mSystems.00543-20-sf006.tif]

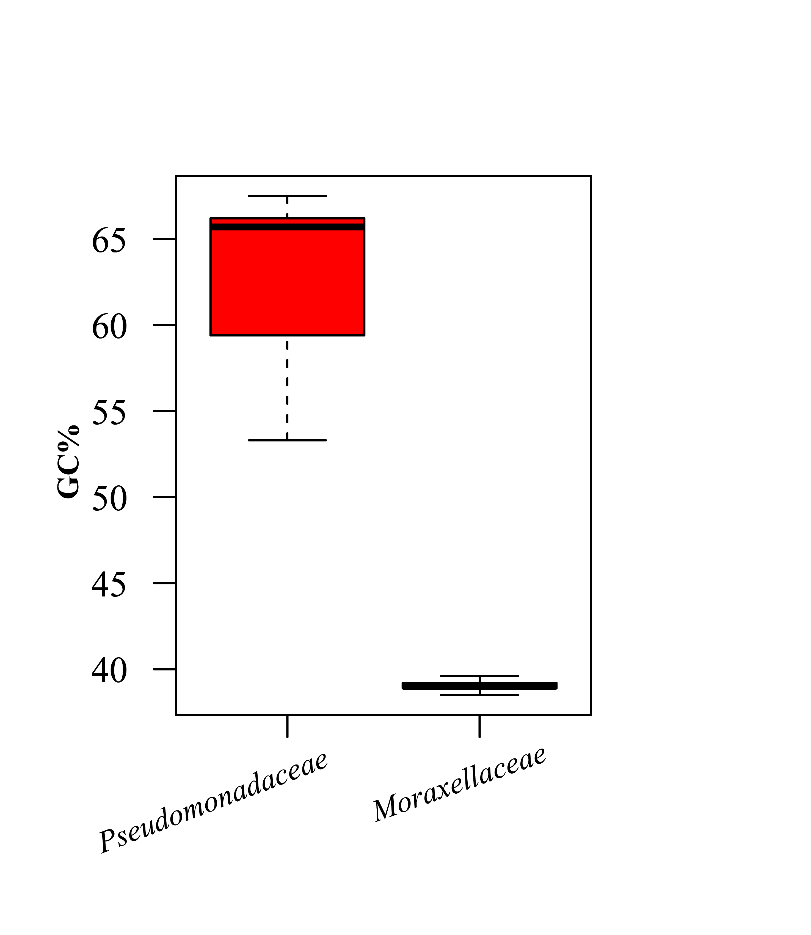


**a**


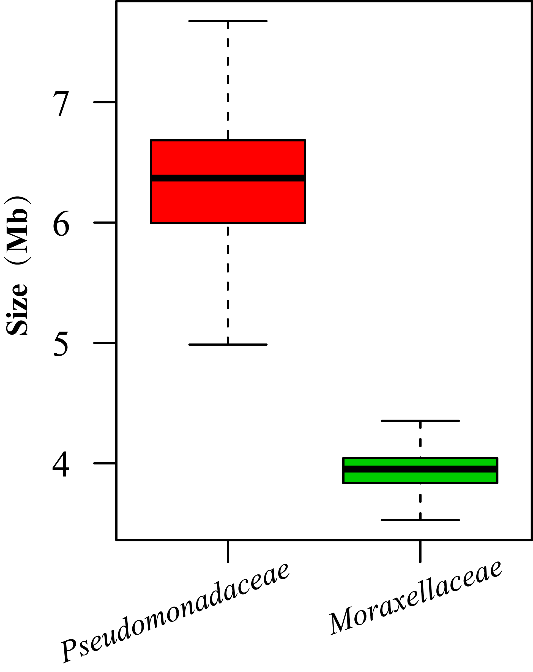


**b**

**Figure S7**

Supplement: FIG S7 [file mSystems.00543-20-sf007.docx]
